# Neighborhood Vulnerability and the Consumer Food Environment in an Urban Area

**DOI:** 10.3390/ijerph22020303

**Published:** 2025-02-18

**Authors:** Cecilia Craveiro, Mariana Lopes, Patricia Freitas, Aline Lopes

**Affiliations:** 1 Grupo de Pesquisa de Intervenções em Nutrição, Departamento de Nutrição, Campus I, Centro de Ciências da Saúde, Universidade Federal da Paraíba, Cidade Universitária, João Pessoa 58051-900, PB, Brazil; ceciliafc23@gmail.com (C.C.); marianalopes.ufpb@gmail.com (M.L.); 2 Grupo de Pesquisa de Intervenções em Nutrição, Universidade Federal dos Vales do Jequitinhonha e Mucuri, Nutrition Building JK Campus, Highway BR 367, Km 583, s/n, Alto da Jacuba District, Diamantina 39100-000, MG, Brazil; patpfreitas@gmail.com; 3 Grupo Pesquisa de Intervenções em Nutrição, Departamento de Nutrição, Universidade Federal de Minas Gerais, Escola de Enfermagem, de 190 Alfredo Balena Avenue, Room 316, Santa Efigênia, Belo Horizonte 30130-100, MG, Brazil

**Keywords:** access to healthy foods, public policies, socioeconomic disparities in health, fruit, vegetables

## Abstract

The consumer food environment is an important medium for understanding complex interactions regarding food consumption, health outcomes and social vulnerability. We aimed to analyze the diversity, variety and quality of natural and ultra-processed foods in a Brazilian metropolis. We performed a cross-sectional study, analyzing food stores within a buffer (1600 m) area around 18 randomly selected Health Promotion Program units. We used descriptive analyses and regression models, adjusted by the area’s population, to examine associations of consumer food environment variables with the health vulnerability (HVI) of the territory and store type. Low HVI areas had higher fruits and vegetables variety adequacy and better quality, when compared to medium and high/very high HVI areas (*p*-value < 0.001 and *p*-value = 0.001). Supermarkets in low HVI areas had almost twice the prevalence of adequate vegetable variety (65.2% vs. 33.3% in high/very high HVI areas, *p*-value = 0.005). Adjusted by population, areas with high/very high HVI had lower odds of adequate fruit variety when compared to low HVI areas (OR = 0.06; CI 95% = 0.01–0.44; *p*-value = 0.006). Although consumer preference is important in food acquisition, disparities in quality, diversity and variety within the consumer food environment could lead to difficulties in access to healthy options for vulnerable populations.

## 1. Introduction

In the food system framework, conceptualized by the High-Level Panel of Experts, the food environment overlaps with consumer behavior, food supply chains and diets, which all influence and are influenced by the food environment. This relation also entails cultural aspects, food preferences and socio-political circumstances, all coordinated with and by policy and governance [1]. These complex systems interact to supply food to consumers, in diverse contexts. Within this, the consumer food environment is related to the availability, quality, diversity, publicity and price of food products inside stores [2].

Studies have been carried out in countries with different levels of inequality, and aimed to understand the relations between food environments, inequality and health-related outcomes [2,3,4,5]. Vulnerability and inequality are multifaceted themes, with food security and health repercussions. It is estimated that one in three people do not have adequate access to food, meaning that 3 billion people do not have access to a healthy diet [6,7].

Difficulties in accessing stores that sell healthy foods were observed in low-income neighborhoods, where fast food chains and convenience stores were more prevalent, while neighborhoods with a lower prevalence of inequality had more stores associated with healthy consumption, such as supermarkets [4]. Communities where inequality was higher tended to have higher access to unhealthy food, while the same was not true for neighborhoods with lower levels of inequality [8,9]. More vulnerable areas also tend to have fewer natural or minimally processed foods such as fruits and vegetables (FV) [10] and higher prices of healthy items, which leads to a harmful food environment [11].

However, some of these studies were limited to investigating the community food environment. Exploration of the consumer environment in areas with health vulnerability is minimal, and also sometimes restricted to countries with lower levels of inequality than Brazil and other middle- and low-income countries [10]. Nonetheless, the investigation of both food environment aspects—community and consumer—as described by Glanz et al. [2] is important to fully understand the dynamic that affects eating behaviors and health. In Brazil, neighborhoods with lower vulnerability tended to have easier access to healthy food stores, and more disadvantaged neighborhoods had fewer of those stores [8,9,12]. However, Brazilian studies are sometimes divergent, indicating that the country has cultural peculiarities that must be explored. Therefore, the consumer food environment tends to be overlooked by policymakers as a possible site for change and public policy intervention [13].

The present study is relevant because although the analysis of the location and type of establishment is important, it may not capture the full scope of the individual’s food choices in the food environment, as other aspects, such as the quality and variety of food, are also pertinent. The current rapid changes occurring in food environments call for a more direct analysis of products being sold, which is not possible when looking only at the community food environment. To analyze the consumer food environment is important to expand possible public policies toward improving food quality and reducing the prevalence of chronic non-communicable diseases, which were responsible for 47.8% of deaths in Belo Horizonte in 2020 [14].

The main objective of this study is to assess the food environment—community and consumer—combined with the variables type of store, variety, diversity and quality, in food stores in neighborhoods with different levels of health vulnerabilities in a Brazilian metropolis.

## 2. Materials and Methods

### 2.1. Study Design and Setting

This is a cross-sectional study, carried out using data collected in 2018 in a previous longitudinal study that audited food stores to investigate the food environment in the areas of the Health Academy Program (Programa Academia da Saúde—PAS, in Portuguese) in Belo Horizonte, Minas Gerais, Brazil. More details can be seen in previous studies [15].

Belo Horizonte is a metropolis with over 2 million inhabitants, which are distributed across 9 administrative regions and 3833 census tracts, which is the smallest area used to plan and collect statistical and census data [16]. Although it was initially planned, urban expansion took place at a much faster rate than expected, leading the city to greater levels of inequality [3]. Therefore, with the goal of reducing access inequality by redirecting resources, the city classifies its census tracts according to the health vulnerability index (HVI), which varies from low to very high [17].

PAS units are distributed throughout the metropolis. PAS is a national public health service that enhances individual and collective health care. In Belo Horizonte, there are currently 83 PAS units, with over 14,000 users. Its main goal is health promotion, prevention and recovery, through physical exercise, nutritional guidance and leisure activities with qualified professionals through specific activities and interventions in mainly more vulnerable areas (medium, high and very high HVI risk), by creating adequate and healthy environments [18,19].

### 2.2. Study Sample

The sampling process of the longitudinal study that preceded this work was carried out in 2012. At the sampling time, 50 PAS units were operating in the municipality. However, to fit in with our inclusion criteria, they had to be open in the mornings and to be located in census tracts with medium, high and very high HVI [20]. Exclusion criteria were the participation in intervention studies in the previous 24 months (*n* = 2). Therefore, of the 42 eligible units, 2 units were drawn from each of the 9 administrative regions, through a simple conglomerate sample, stratified by the administrative regions and totaling 18 units. These units were representative of the municipality, with 95% confidence and <1.4% error. More information about methods and sampling can be seen in a previous publication [20].

To analyze the consumer food environment, we audited food stores and open-air food markets that sold FV located within 1600 m (1 mile) of each PAS unit, as this is a commonly used search diameter and it is shown as a walkable distance [10]. In 2013, food stores were selected from data provided by the municipal government and by the Secretaria Municipal Adjunta de Arrecadação, in Portuguese. After mapping all locations in the selected radius, a direct search was used to add those with no formal registration (in Brazil, the National Register of Legal Entities) and those that were recently opened. In 2018, the audit started from the initial database, excluding establishments that had closed and including those who opened, by direct search [15].

### 2.3. Data Collection

All stores were audited by trained dietitians. We investigated the consumer food environment regarding healthy and unhealthy food: natural FV (diversity, variety and quality) and UPFs (ultra-processed foods) (availability and variety).

All food stores in the consumer food environment were assessed using an adapted version of the ESAO-s tool, constructed for the Obesogenic Environment Study in Sao Paulo, Brazil, and validated for the Brazilian setting and adapted for the 10 most frequently purchased fruits (bananas, oranges, papaya, watermelon, apples, mangoes, pineapple, tangerines, grapes and melons) and vegetables (pumpkins, chayote, tomatoes, carrots, lettuce, zucchini, cabbage, beetroot, kale and okra) in the municipality. Tubers and roots were not considered in the vegetable assessments, as they were classified under a separate vegetable category [8,21].

The UPF evaluated included the four most consumed in Brazil, which are regular soda, fruit-flavored drinks and juice or nectars with added sugar, chocolate sandwich cookies and corn chips [8,21,22].

The availability of FV and UPFs was evaluated by the presence of at least one item. We determined the variety of FV by the number of different types of items (e.g., iceberg lettuce, green leaf lettuce, red leaf lettuce) and of UPFs by different brands and flavors. The quality was assessed based on whether most of the food was withered, bruised, overripe or old-looking. We also observed if there was any UPF item in the FV area [23].

### 2.4. Study Variables

#### 2.4.1. Outcome: Diversity, Variety and Quality of FV and UPFs

Consumer environment variables were the outcomes of our study and included diversity, variety and quality of the 10 most consumed fruits and vegetables in Belo Horizonte, as well as the diversity and variety of the 4 most consumed UPF products, and presence or absence in the FV area.

Definitions and cutoff points are present in Table 1.

#### 2.4.2. Explanation of Main Interest: Health Vulnerability Index

The HVI (Índice de Vulnerabilidade da Saúde—IVS, in Portuguese) is calculated from sociodemographic variables, and it points to the more vulnerable areas in the city, for resource redistribution. The index was initially conceived in Belo Horizonte and was first calculated in 1998. The last measure (2012) used 8 indicators (Table A1), selected in a participatory manner, by specialists, technicians and academics experts. Each indicator was transformed into values from 0 to 1, and weighed in the same participative manner [17].

The geographic unit analyzed was the census tract, and the results can indicate low, medium, high or very high vulnerability risk. Due to the low number of areas with very high HVI, we combined high and very high regions, classifying them as high/very high. A region is classified as low HVI when its HVI value is lower than the medium HVI, which in turn are those regions with an HVI value of 0.5 standard deviation (SD) around the mean. High-risk HVI regions have values above the medium HVI, up to 1.5 standard deviations above the average, and regions with very high risk are those with values exceeding the high HVI [17]. For the purposes of this study, the vulnerability of the establishment’s census tract was considered.

#### 2.4.3. Other Explanatory Variables of Interest: Food Store Types

In Brazil, food stores can offer a multitude of options, and the food store type is one of the factors that can determine the main product sold. During the audit, stores were classified according to type in (i) supermarkets; (ii) FV-specialized markets including open-air food markets and (iii) local stores (local grocery stores, convenience stores, delis and bakeries). In Minas Gerais, according to food desert mapping [24], supermarkets are considered mixed establishments, where you can buy both natural and UPF foods, FV-specialized stores sell mostly healthy items, and local stores are places with higher prevalence of UPFs. Therefore, store types were also used to assess possible differences in the consumer food environment.

#### 2.4.4. Adjustment Covariate

The data were adjusted by the total population of the census tracts of stores, which was sourced from the 2010 Brazil Census [16]. The number of people in an area could be associated with the distribution of food stores because the number of food stores tends to be higher in more densely populated areas [12].

### 2.5. Data Analysis

All analyses were performed with the statistical software package Stata/SE version 14 [25] and the statistical significance was set at 5%.

Characteristics of the consumer environment (diversity, variety and quality for FV; availability and variety for UPFs) were described by frequencies, mean and confidence interval (CI) by HVI level.

The logistic regression-based approach was implemented in order to determine the association between the outcomes, the different levels of HVI and food store types adjusted by population.

### 2.6. Ethics

This study does not involve human data. However, those responsible for the commercial establishments authorized the audit and signed a written informed consent form. The protocol was approved by the Ethics Committee University (0537.0.0203.000-11) and the City Hall (0537.0.0203.410-11A) and conducted according to the guidelines of the Declaration of Helsinki.

## 3. Results

Our study analyzed 258 stores. Of those, 48 were supermarkets, 168 were specialized FV stores and 42 were classified as local stores; 47.9% of supermarkets were located in areas of low HVI, while 12.5% were located in high/very high HVI areas; and 36.9% of FV-specialized stores were present in regions with low HVI and 17.3% were in high/very high HVI regions. As for local stores, their presence was higher in regions with high/very high HVI regions than in regions with low HVI (26.2% vs. 19.1%) (Table A2).

Regarding the consumer environment variables for FV (diversity, variety and quality), in most stores, diversity (65.1%) and quality (81.5%) of fruits were adequate, while 21.7% had adequate variety (Table 2). Most stores also had adequate vegetable diversity (57.8%) and quality (75.3%), while 24.4% had adequate variety. There was a significant difference in the variety and quality of FV according to the HVI. Low HVI areas had a higher share of adequate FV variety and better quality, when compared to their counterparts (*p*-value < 0.001 and *p*-value = 0.001). As for UPF items, no differences between HVI areas were found regarding the variety, availability and presence of UPFs in the FV area.

Regarding the fruit variety, low HVI areas had 34.4% of stores with adequate variety, while 2.2% of stores in high/very high HVI areas had adequate variety (*p*-value < 0.001). On fruit quality, 94.1% of stores in low HVI areas had adequate quality vs. 69.6% in high/very high HVI areas (*p*-value = 0.001).

Regarding the vegetable variety and quality, 10.9% of stores that had adequate vegetable variety were located in high/very high HVI areas (*p*-value < 0.001), while 38.7% of stores that had adequate variety were in low HVI areas. In high/very high HVI areas, 60% of stores had adequate quality; and in low HVI areas, the prevalence was 88.1% (*p*-value = 0.001).

In Table 3, Table 4 and Table 5, we have stratified our analysis by store type. Supermarkets located in low HVI areas when compared to supermarkets in high/very high HVI areas had almost twice the prevalence of adequate vegetable variety (65.2% vs. 33.3%, *p*-value = 0.005) (Table 3).

As for FV-specialized stores (Table 4), differences were found in vegetable quality and fruit quality and variety according to the HVI; 33.9% of FV-specialized stores with adequate fruit variety were located in low HVI areas, while none of the stores in high/very high areas had adequate variety (33.9% vs. 0.0%, *p*-value = 0.002). Regarding fruit quality, low HVI areas had a higher share of stores with adequate quality, when compared to FV-specialized stores in areas with high/very high HVI (94.6% vs. 68.1%, *p*-value = 0.001).

A similar situation was found for vegetable quality, where the prevalence of FV-specialized stores with adequate quality vegetables was higher in low HVI areas when compared to their counterparts (92.7% vs. 76.3% at medium HVI areas; and 92.7% vs. 69.0% at high/very high HVI areas, *p*-value = 0.014). No statistical difference was found between HVI areas in vegetable diversity and variety.

No differences between consumer environment variables and HVI were observed in local stores (Table 5).

Table 6 shows the results after adjustment by population. Regardless of the type of store, census tracts with high/very high HVI had lower chances of adequate fruit variety when compared to low HVI areas (OR = 0.06; CI 95% = 0.01–0.44; *p*-value = 0.006). Similar results were observed for fruit quality. Stores located in medium HVI areas (OR = 0.26; *p*-value 0.011) and in high/very high HVI areas (OR = 0.18; CI 95% = 0.06–0.5; *p*-value = 0.004) had lower odds of good fruit quality than stores in low HVI areas.

Still regarding fruits, local stores, when compared to supermarkets, had unlikely odds of adequate fruit diversity (OR = 0.14; CI 95% 0.05–0.36; *p*-value < 0.001) and variety (OR = 0.06; CI 95% 0.01–0.53; *p*-value = 0.011), as well as lower odds of adequate fruit quality (OR = 0.07; CI 95% 0.01–0.59; *p*-value = 0.014). FV-specialized stores, when compared to supermarkets, also had lower chances of good fruit quality (OR = 0.09; CI 95% 0.01–0.72; *p*-value = 0.023).

When we looked at vegetables in different HVI regions, independent of store type and adjusted by population, variety was less likely to be adequate in locations with medium HVI (OR = 0.39; CI 95% 0.20–0.78; *p*-value = 0.008) and high/very high HVI areas (OR = 0.22; CI 95% 0.09–0.5; *p*-value = 0.006). Vegetable quality also had lower odds of being good in medium HVI areas (OR = 0.38; CI 95% 0.17–0.85; *p*-value 0.018) and in high/very high HVI areas (OR = 0.22; CI 95% 0.09–0.55; *p*-value 0.001), when compared to low HVI areas.

When looking at the types of stores, we found that when compared to supermarkets, local stores had lower odds of adequate vegetable variety (OR = 0.09; CI 95% 0.02–0.45; *p*-value 0.003) and diversity (OR = 0.23; CI 95% 0.09–0.57; *p*-value 0.001), but this was not observed for vegetable quality.

Table 7 shows the results for UPFs, which were available in all stores, regardless of type. The UPF variety was lower compared to supermarkets in stores specializing in fruits and vegetables (OR = 0.05, *p*-value < 0.001).

## 4. Discussion

In our study, the consumer environment variables differed according to vulnerability levels in a metropolis in Brazil, which persisted according to store types. Areas with high/very high-risk vulnerability levels had lower fruit and vegetable varieties and quality when compared to areas with low vulnerability. However, there was no significant difference for UPFs.

Although we found differences in the community food environment in Belo Horizonte, these disparities went even further when we looked into the consumer food environment. Areas with high/very high vulnerability risk had fewer supermarkets and fewer FV-specialized stores than areas with low vulnerability risk, which was aggravated when we looked at variables in the consumer food environment. Furthermore, the fact that there was no significant difference for UPFs could negatively impact those individuals with fewer healthy options available. These differences could lead to worse health outcomes for these vulnerable populations [5,7].

More specifically, we found differences in the variety and quality of fruits and vegetables based on the vulnerability of food store locations. Areas with high/very high vulnerability risk had lower odds of offering fruits and vegetables with adequate variety and good quality compared to areas with low vulnerability risk. For fruits, the results were even worse, as the odds of finding good quality and adequate variety were lower not only by vulnerability but also by store type, highlighting that access to fruits is even more difficult than access to vegetables. This issue is compounded by the fact that fruits are generally more expensive than vegetables [26].

The better outcomes observed for vegetables could be due to Brazilians’ habit of including vegetables in their main meals, which impulses supply and demand for vegetables in food stores [27]. In light of this, we can only expect healthy individual choices if the environment supports them with accessible and affordable options [28]. In the context of this study, vulnerable regions do not provide adequate quality and variety of fruits and vegetables, alongside a persistent presence of UPFs, regardless of the vulnerability of the territory, which could make it difficult to implement and maintain healthy eating habits. Current food environments are therefore exploited by the food industry, through “supply-driven consumption of unhealthy food” [29].

Further analysis revealed that local stores, compared to supermarkets, were less likely to offer fruits with adequate variety, quality and diversity. Borges et al. [30] found that supermarkets offered more natural foods, and also that local stores had more barriers to healthy eating than other types of stores. This is also aggravated by the fact that most supermarkets in our study were located in regions with low HVI. In a longitudinal analysis conducted by Justiniano et al. [31], it was also shown that over ten years, there was a reduction in stores offering healthy options in Belo Horizonte, particularly between 2015 and 2018. An analysis performed in Mexico also noted that regions with a higher prevalence of fast-food outlets and convenience stores were associated with unhealthy dietary patterns, while also being linked to lower socioeconomic status [32].

When compared to supermarkets, FV-specialized stores were less likely to offer good quality fruit. This could be due to a gradual change observed in Brazil and other countries. The supermarket sector has been increasing its share of retail food sales, while the demand for quality in supermarkets is on the rise, which slowly has been distancing the population from more sustainable and biodiverse ways of acquiring food, such as agro-ecological markets, which also celebrate traditional and community knowledge [33]. Small producers also face difficulties in selling their fresh products and expanding their production, as middlemen and supermarket chains retain profits and exclude smallholders from decision-making [34,35].

However, we cannot ignore that nowadays most of the Brazilian population shops at supermarkets [15,36], and therefore it should be noted that 100% of supermarkets and local stores had UPF items available. The growth of UPF markets in low- and middle-income countries (LMICs) was observed by Baker et al. [37], who said that urbanization, economic development, higher income and changes in work structures and demographics all lead to higher variety and availability of UPFs in these countries. Nevertheless, high-income countries also have similar circumstances. A study in Ireland found that supermarkets offered unhealthier options at higher vantage points on shelves [38], and Baker et al. [37] also found that although sales have been increasing in LMICs, regions such as Western Europe and North America still account for the largest share of the market.

Further environmental changes were explored by De Bruin, Dengerink and Van Vliet [39], highlighting how urbanization is responsible for higher food demand, changes in food preferences and purchasing power, formalization and complexification of value chains, while increasing the pressure on food systems [34]. Swinburn et al. [27] also explored how UPF production has a higher impact both on climate change and obesity, impacting the food systems at different scales. We emphasize the importance of this perspective in our findings, since UPFs were found in most stores, regardless of vulnerability or store type. However, less vulnerable populations also had healthier and higher-quality options available. More vulnerable communities are already exposed to worse health services and higher prevalence of NCD. These issues are compounded by reduced access to healthy food [9,13,32].

Our findings highlight the urgent need for action in food environments and in food systems in a comprehensive and systematic manner, with political and ecological interventions. Policymakers and decision-makers, as well as civil society, should focus on legislation and regulation to tackle problems caused by our current food system. In the food environment, efforts should concentrate on regulating food marketing and frontal labeling [36]. In food production and retail, initiatives should focus on tax reduction for natural foods, implementation of UPFs and sugar taxes, improving food composition, and regulation for supply chains, in an attempt to reduce costs for small producers [35,37].

In that regard, this article comes at an opportune time, as in 2023, the first National Food Supply Policy was announced in Brazil, with a detailed plan of action released in 2024. It aims to connect food production, food supply and availability in an effort to promote healthier and more sustainable food options to the population, through family agriculture supply and commercial centers, connected with pesticide reduction policies, among others [40]. Belo Horizonte, where we conducted our study, is one of the cities that count with public facilities that provide access to healthy food, such as municipal fruits and vegetables markets, community kitchens and agro-ecological food production supported by the municipality, which represents an initial effort to act on social and nutritional disparities. In this context, we would also like to emphasize how the study of food availability and variety is important to promote healthy and sustainable food systems through the strengthening of biodiversity, which is paramount to food security and nutrition, while also pointing to decision-makers where food security and nutrition policies are most needed [41].

Food security and nutrition policies must be associated with health policies. In this sense, we elected the Health Academy Program (PAS) as a starting point for mapping out food stores, as the program is an instrument of Brazil’s Health Unified System and could facilitate and optimize future interventions, both in the community and on individual levels.

There is a limitation in the data collected for open-air food markets, since the tool was only applied to natural food and not UPF items, which could be present in those markets. However, open-air food markets are scarce in Belo Horizonte, so the limitation is minimal. In addition, variables such as price, promotion, affordability, convenience and desirability are equally important to the comprehension of the consumer food environment and were not included in our study, which could limit the full understanding of the food environments we analyzed. Future research could further explore these dimensions to complement our findings. Another limitation of our study is that, since its data collection in 2018, food environments have gone through changes, which have drastically transformed how consumers interact with them. Food environments have become more dynamic and influenced by the digital food environment, with less-studied value chains, characteristics not captured in our study. However, this does not invalidate our study and data.

Our study is one of the first in Brazil to analyze the consumer food environment in relation to the vulnerability of the area, highlighting possible socioeconomic disparities in the food environment. It is also one of the few studies in Brazil to perform audits directly in the store, while also searching for stores that were not present in the consulted databases. In addition, we applied a previously validated tool for Brazil’s reality, which strengthens our findings.

## 5. Conclusions

In Belo Horizonte, areas with high/very high-risk vulnerability levels had lower fruit and vegetable varieties and quality when compared to areas with low vulnerability. However, there was no significant difference for UPFs. These disparities in the consumer food environment could lead to difficulties in accessing healthy options, while simultaneously promoting unhealthy items. The differences observed by store type confirm that the community food environment is unequal, but our study also shows that disparities go even further in the consumer food environment. With this in mind, we affirm that interventions in the food environment need to address not only the availability of stores that sell healthy items but also the disparities, in a more targeted manner, such as in the variety and quality of products. We recognize that consumer choice plays an important part in food acquisition, but they are also influenced and molded by the environment. So, it is necessary to promote a food environment that enables and supports healthier choices that could lead to positive impacts on planetary health, especially for vulnerable populations with limited access to healthy food.

## Figures and Tables

**Table 1:** Variables assessed within the consumer food environment. Belo Horizonte, Minas Gerais, Brazil, 2018.

Variable		Measure	Evaluation
Fruits	Diversity	Number of fruits among the 10 investigated items	Inadequate: below median Adequate: above median
	Variety of certain fruits	Number of different types of fruit within each kind (e.g., apple—green apple, gala apple, fuji apple) among the 10 items investigated	Inadequate: <fourth quartile of the total number of available fruit varieties Adequate: fourth quartile of the total number of available fruit varieties
	Quality	Subjective rating (bruised, old-looking, overripe, or spotted) of the 4 most consumed fruits in the city	Good: ≥75% of the investigated fruits were evaluated as good Bad: ≥25% of the investigated fruits were evaluated as bad
Vegetables	Diversity	Number of vegetables among the 10 investigated items	Inadequate: below median Adequate: above median
	Variety of certain vegetables	Number of different types of vegetable within each kind (e.g., green cabbage, red cabbage) among the 10 items investigated	Inadequate: <fourth quartile of the total number of available vegetable varieties Adequate: fourth quartile of the total number of available vegetable varieties
	Quality	Subjective rating (bruised, old-looking, overripe, or spotted) of the 4 most consumed vegetables in the city	Good: ≥75% of the investigated vegetables were evaluated as good Bad: ≥25% of the investigated vegetables were evaluated as bad
UPF	Availability	Availability of any UPF	Not available: no products were found Available: ≥1 product was found
	Variety	The number of different brands and flavors among the 5 items investigated	No variety: below median <1 * Any variety: above median ≥1
	In fruits and vegetables area	Presence or absence in fruits and vegetables areas in stores	Presence: UFP found in the area Absence: no UPF found in the area

Notes: FV = Fruits and Vegetables; UPFs = Ultra-processed foods. * As the median was one, we chose to use “no variety” when no UPFs were available. Adapted from De Freitas et al. [23].

**Table 2:** Consumer environment variables according to the Health Vulnerability Index. Belo Horizonte, Minas Gerais, Brazil 2018.

Variables		Total (%)	HVI *			*p*-Value ***
			Low (%)	Medium (%)	High/Very High (%)	
Fruits						
Diversity ^a^	Adequate	65.1	69.9	63.9	58.7	0.396
Variety ^b^		21.7	34.4	19.3	2.2	**<0.001**
Quality ^c^		81.5	94.1	77.1	69.6	**0.001**
Vegetables						
Diversity ^a^	Adequate	57.8	58.1	58.0	56.5	0.983
Variety ^b^		24.4	38.7	18.5	10.9	**<0.001**
Quality ^c^		75.3	88.1	72.0	60.0	**0.001**
Ultra-processed foods **						
Variety	Any variety	46.9	44.1	51.3	41.3	0.410
	No variety	53.1	55.9	48.7	58.7	
Availability	Available	59.7	59.1	61.3	56.5	0.844
	Not available	40.3	40.9	38.7	43.5	
In fruits and vegetables area	Presence	46.4	45.2	50.0	39.1	0.441
	Absence	53.6	54.8	50.0	60.9	

* HVI = Health Vulnerability Index; HVI Classification: Low < Medium HVI; Medium HVI = mean ± 0.5 SD; High/Very high HVI = medium HVI upper limit + 1 SD/above high HVI. ** No data were collected for UPFs at open-air food markets. *** *p*-value < 0.05 was considered significant, highlighted in bold. ^a^ Adequate diversity: above the median. ^b^ Adequate variety: fourth quartile of the total number of available fruit varieties. ^c^ Adequate quality: ≥75% of the investigated fruits were evaluated as good.

**Table 3:** Consumer environment variables of fruits, vegetables and ultra-processed foods in supermarkets. Belo Horizonte, Minas Gerais, Brazil, 2018.

Variables		Total (%)	HVI *			*p*-Value **
			Low (%)	Medium (%)	High/Very High (%)	
Fruits						
Diversity ^a^	Adequate	75.0	82.6	63.2	83.3	0.308
Variety ^b^		35.4	43.5	31.6	16.7	0.428
Quality ^c^		97.2	95.7	100.0	100.0	0.574
Vegetables						
Diversity ^a^	Adequate	62.5	69.6	57.9	50.0	0.588
Variety ^b^		41.7	65.2	15.8	33.3	**0.005**
Quality ^c^		75.0	82.6	68.4	66.7	0.504
Ultra-processed foods						
Variety	Any variety	87.5	82.6	94.7	83.3	0.470
	No variety	12.5	17.4	5.3	16.7	
Availability	Available	100.0	100.0	100.0	100.0	-
	Not available	0.0	0.0	0.0	0.0	
In fruits and vegetables area	Presence	81.3	78.3	84.2	83.3	0.878
	Absence	18.8	21.7	15.8	16.7	

* HVI = Health Vulnerability Index; HVI Classification: Low < Medium HVI; Medium HVI = mean ± 0.5 SD; High/Very high HVI = medium HVI upper limit + 1 SD/above high HVI. ** *p*-value < 0.05 was considered significant, highlighted in bold. ^a^ Adequate diversity: above the median. ^b^ Adequate variety: fourth quartile of the total number of available fruit varieties. ^c^ Adequate quality: ≥75% of the investigated fruits were evaluated as good.

**Table 4:** Consumer environment variables of fruits, vegetables and ultra-processed foods in FV stores. Belo Horizonte, Minas Gerais, Brazil, 2018.

Variables		Total (%)	HVI *			*p*-Value ***
			Low (%)	Medium (%)	High/Very High (%)	
Fruits						
**Diversity ^a^**	**Adequate**	71.4	67.7	75.3	69.0	0.585
**Variety ^b^**		22.6	33.9	22.1	0.0	**0.002**
**Quality ^c^**		79.4	94.6	75.0	68.1	**0.001**
Vegetables						
**Diversity ^a^**	**Adequate**	63.7	54.8	67.5	72.4	0.170
**Variety ^b^**		24.4	32.3	23.4	10.3	0.073
**Quality ^c^**		80.6	92.7	76.3	69.0	**0.014**
Ultra-processed foods **						
**Variety**	**Any variety**	27.4	24.2	32.5	20.7	0.373
	**No variety**	72.6	75.8	67.5	79.3	
**Availability**	**Available**	38.1	38.7	40.3	31.0	0.678
	**Not available**	61.9	61.3	59.7	69.0	
**In fruits and vegetables area**	**Presence**	31.7	26.4	36.8	27.6	0.399
	**Absence**	68.3	73.6	63.2	72.4	

* HVI = Health Vulnerability Index; HVI Classification: Low < Medium HVI; Medium HVI = mean ± 0.5 SD; High/Very high HVI = medium HVI upper limit + 1 SD/above high HVI. ** No data were collected for UPFs at open-air food markets. *** *p*-value < 0.05 was considered significant, highlighted in bold. ^a^ Adequate diversity: above the median. ^b^ Adequate variety: fourth quartile of the total number of available fruit varieties. ^c^ Adequate quality: ≥75% of the investigated fruits were evaluated as good.

**Table 5:** Consumer environment variables of fruits, vegetables and ultra-processed foods in local stores. Belo Horizonte, Minas Gerais, Brazil, 2018.

Variables		Total (%)	HVI *			*p*-Value **
			Low (%)	Medium (%)	High/Very High (%)	
Fruits						
Diversity ^a^	Adequate	28.6	50.0	26.1	18.2	0.294
Variety ^b^		2.4	12.5	0.0	0.0	0.113
Quality ^c^		70.0	83.3	65.2	72.7	0.671
Vegetables						
Diversity ^a^	Adequate	28.6	50.0	26.1	18.2	0.294
Variety ^b^		4.8	12.5	4.4	0.0	0.446
Quality ^c^		53.9	66.7	60.9	30.0	0.208
Ultra-processed foods						
Variety	Any variety	78.6	87.5	78.3	72.7	0.740
	No variety	21.4	12.5	21.7	27.3	
Availability	Available	100.0	100.0	100.0	100.0	-
	Not available	0.0	0.0	0.0	0.0	
In fruits and vegetables area	Presence	61.9	75.0	65.2	45.5	0.377
	Absence	38.1	25.0	34.8	54.5	

* HVI = Health Vulnerability Index; HVI Classification: Low < Medium HVI; Medium HVI = mean ± 0.5 SD; High/Very high HVI = medium HVI upper limit + 1 SD/above high HVI. ** *p*-value < 0.05 was considered significant. ^a^ Adequate diversity: above the median. ^b^ Adequate variety: fourth quartile of the total number of available fruit varieties. ^c^ Adequate quality: ≥75% of the investigated fruits were evaluated as good.

**Table 6:** Results of diversity, variety and quality of fruits and vegetables according to different types of food stores and HVI, 2018.

	Variables	Diversity				Variety		Quality		
		Estimative	CI (95%)	*p*-Value	Estimative	CI (95%)	*p*-Value	Estimative	CI (95%)	*p*-Value ***
F r u i t s	HVI *									
	Low	ref.	-	-	ref.	-	-	ref.	-	-
	Medium	0.90	0.47–1.72	0.756	0.22	0.31–1.24	0.175	**0.26**	**0.09–0.73**	**0.011**
	High/Very high	0.77	0.03–1.74	0.536	**0.06**	**0.01–0.44**	**0.006**	**0.18**	**0.06–0.57**	**0.004**
	Store type									
	Supermarkets	ref.	-	-	ref.	-	-	ref.	-	-
	FV-specialized store	0.87	0.41–1.84	0.720	0.66	0.31–1.38	0.269	**0.09**	**0.01–0.72**	**0.023**
	Local stores	**0.14**	**0.05–0.36**	**<0.001**	**0.06**	**0.01–0.53**	**0.011**	**0.07**	**0.01–0.59**	**0.014**
	Total population **	1.00	0.99–1.00	0.494	0.99	0.99–1.00	0.400	0.99	0.99–1.00	0.746
V e g e t a b l e s	HVI *									
	Low	ref.	-	-	ref.	-	-	ref.	-	-
	Medium	1.19	0.65–2.19	0.564	**0.39**	**0.20–0.78**	**0.008**	**0.38**	**0.17–0.85**	**0.018**
	High/Very high	1.21	0.55–2.63	0.639	**0.22**	**0.08–0.64**	**0.006**	**0.22**	**0.09–0.55**	**0.001**
	Store type									
	Supermarkets	ref.	-	-	ref.	-	-	ref.	-	-
	FV-specialized store	1.02	0.52–2.02	0.951	0.52	0.26–1.07	0.075	1.69	0.76–3.75	0.201
	Local stores	**0.23**	**0.09–0.57**	**0.001**	**0.09**	**0.02–0.45**	**0.003**	0.54	0.21–1.41	0.211
	Total population **	0.99	0.99–1.00	0.781	1.00	0.99–1.00	0.435	1.00	0.99–1.00	0.737

* HVI = Health Vulnerability Index; HVI Classification: Low < Medium HVI; Medium HVI = mean ± 0.5 SD; High/Very high HVI = medium HVI upper limit + 1 SD/above high HVI. ** Total population from each census tract was sourced from the 2010 Brazil Census. *** *p*-value < 0.05 was considered significant, highlighted in bold.

**Table 7:** Results of diversity and variety of ultra-processed food according to different HVI levels and types of food stores, 2018.

Variables	Diversity			Variety		
	Estimative	CI (95%)	*p*-Value	Estimative	CI (95%)	*p*-Value ***
HVI *						
Low	ref.	-	-	ref.	-	-
Medium	1.07	0.51–2.23	0.862	1.38	0.69–2.75	0.366
High/Very high	0.71	0.27–1.92	0.502	0.76	0.31–1.87	0.543
Store type						
Supermarkets	1	-	-	ref.	-	-
FV-specialized stores	1	-	-	0.05	0.02–0.13	**<0.001**
Local stores	1	-	**-**	0.52	0.16–1.64	0.261
Store density **	0.99	0.99–1.00	0.998	1	0.99–1.00	0.418

No data were collected for UPFs at open-air food markets, due to the tool’s limitation. * HVI = Health Vulnerability Index; HVI Classification: Low < Medium HVI; Medium HVI = mean ± 0.5 SD; High/Very high HVI = medium HVI upper limit + 1 SD/above high HVI. ** Store density is calculated by number of stores divided by the population of the census tract. *** *p*-value < 0.05 was considered significant, highlight in bold.

## Data Availability

The data presented in this study are available on request from the corresponding author due to privacy reasons.

## Appendix A

**Table A1:** Health vulnerability index indicators. Belo Horizonte, 2013.

Dimension	Indicator
Sanitation	Percentage of permanent private households with inadequate or no water supply
	Percentage of permanent private households with inadequate or no sewage disposal
	Percentage of permanent private households with inadequate or no waste disposal
Socioeconomic	Ratio of residents per household
	Percentage of illiterate people
	Percentage of private households with per capita income of up to ½ minimum wage
	Average nominal monthly income of person in charge (inverted)
	Percentage of black, brown and Indigenous people

Source: Adapted from Índice de Vulnerabilidade da Saúde [16].

**Table A2:** Distribution of food stores according to HVI. Belo Horizonte, 2013.

Food Store Type	HVI *			Total (n)
	Low (%)	Medium (%)	High/Very High (%)	
Supermarkets	47.9	39.6	12.5	48
FV-specialized stores	36.9	45.8	17.3	168
Local stores	19.1	54.8	26.2	42

* HVI = Health Vulnerability Index; HVI Classification: Low < Medium HVI; Medium HVI = mean ± 0.5 SD; High/Very high HVI = medium HVI upper limit + 1 SD/above high HVI.
